# Follow the Metaplasia: Characteristics and Oncogenic Implications of Metaplasia’s Pattern of Spread Throughout the Stomach

**DOI:** 10.3389/fcell.2021.741574

**Published:** 2021-11-12

**Authors:** José B. Sáenz

**Affiliations:** Division of Gastroenterology, Department of Medicine, Washington University in St. Louis School of Medicine, St. Louis, MO, United States

**Keywords:** metaplasia, *Helicobacter pylori*, atrophy, cancer, inflammation

## Abstract

The human stomach functions as both a digestive and innate immune organ. Its main product, acid, rapidly breaks down ingested products and equally serves as a highly effective microbial filter. The gastric epithelium has evolved mechanisms to appropriately handle the myriad of injurious substances, both exogenous and endogenous, to maintain the epithelial barrier and restore homeostasis. The most significant chronic insult that the stomach must face is *Helicobacter pylori* (Hp), a stomach-adapted bacterium that can colonize the stomach and induce chronic inflammatory and pre-neoplastic changes. The progression from chronic inflammation to dysplasia relies on the decades-long interplay between this oncobacterium and its gastric host. This review summarizes the functional and molecular regionalization of the stomach at homeostasis and details how chronic inflammation can lead to characteristic alterations in these developmental demarcations, both at the topographic and glandular levels. More importantly, this review illustrates our current understanding of the epithelial mechanisms that underlie the pre-malignant gastric landscape, how Hp adapts to and exploits these changes, and the clinical implications of identifying these changes in order to stratify patients at risk of developing gastric cancer, a leading cause of cancer-related deaths worldwide.

## Introduction

### The Stomach as a Microbial Filter

The human stomach represents a unique digestive organ, capable of generating a remarkably acidic environment that serves essential physiologic and metabolic functions. While the stomach is also the source of multiple hormones that regulate satiety and hunger (Murphy and Bloom, 2006; Stengel and Tache, 2012; Stimac et al., 2014), for example, its endocrine function is dwarfed by its distinct exocrine ability to secrete acid. Acid defines the stomach and its purpose in the digestive tract (O'Connor and O'Morain, 2014; Schubert, 2015). From a nutritional standpoint, the ability of the human stomach to consistently maintain a pH less than 2 translates to an effective solubilization of ingested food and the reduction of cations, such as Fe^3+^ to Fe^2+^, for downstream intestinal absorption (Ems et al., 2021).

Perhaps the most fundamental and crucial function of acid is anti-microbial. Gastric acid serves as the primary line of defense against the myriad of microbes that are either ingested or resident to the oropharynx (Giannella et al., 1972). The potent neutralizing ability of gastric acid had long been known and was the impetus for many of the seminal studies that informed our current understanding of gastric physiology (Fourcroy, 1791). Moreover, the gastric mucosa’s tolerance to endogenous acid implies that the gastric epithelium has evolved mechanisms to avoid digesting itself (Wallace, 2008) and to appropriately respond to peptic injury through epithelial restitution (Lacy and Ito, 1984; Sorbye et al., 1988; Paimela et al., 1993), mucus production (Ichikawa, 2011; McColl, 2012), and prostaglandin synthesis (Robert et al., 1979; Konturek et al., 1982; Peskar, 2001).

One could argue that the large vat of hydrochloric acid that is the stomach is unrivaled as a microbial filter. Compared to the more distal intestine, where bacterial densities can reach levels as high as 10^8^ cfu/ml in the cecum, for example (Marteau et al., 2001), the stomach is a bacterial desert, relatively speaking, with bacterial concentrations nearly one million times less dense (Giannella et al., 1972). Indeed, gastric acid eliminates over 99.9% of the micro-organisms that it encounters (Giannella et al., 1972) and represents an insurmountable barrier to chronic colonization for the vast majority of swallowed pathogens. The clinical consequences of raising intra-gastric pH, either through a loss of acid production or pharmacologic inhibition of acid secretion, have been intensely studied, particularly after the introduction of histamine receptor antagonists and, more recently, proton pump inhibitors (PPI) (Sanduleanu et al., 2001; Leonard et al., 2007; Janarthanan et al., 2012; Freedberg et al., 2014; McDonald et al., 2015). While the effects of long-term pharmacologic acid inhibition have been linked to a variety of pathophysiologic processes, ranging from chronic kidney disease to dementia, most of the published literature has relied on retrospective and observational studies that have failed to provide adequate evidence for causation (Vaezi et al., 2017). Of note, acid inhibition has been associated with an increased risk of enteric infections (Leonard et al., 2007; Janarthanan et al., 2012; McDonald et al., 2015), most notably nosocomial *Clostridium difficile* infection (McDonald et al., 2015; Tariq et al., 2017). In addition, gastric acidity has also been shown to promote intestinal microbial homeostasis, with PPI use demonstrating more destabilizing effects than antibiotics (Imhann et al., 2016). It seems biologically plausible that a reduction in one of the gastrointestinal tract’s most potent innate immune defenses could lead to gut dysbiosis, though well controlled prospective trials are still lacking (Villafuerte-Galvez and Kelly, 2018).

### Developmental Demarcations in the Stomach

Broadly speaking, the stomach can be divided into two functionally distinct regions (Ban, 2013). The gastric corpus (or body) accounts for approximately two-thirds of the gastric surface area and is primarily responsible for generating gastric acid. The acid-producing, or oxyntic, glands of the corpus are lined by various epithelial cell types but are primarily defined by two distinct lineages, the parietal cell and the chief cell (Saenz and Mills, 2018). Oxyntic glands generate acidic gastric juice, primarily composed of hydrochloric acid, bicarbonate, and proteolytic enzymes, that aid in digesting food and creating a microbial barrier. The parietal cell secretes hydrochloric acid via its H^+^/K^+^ ATPase (Engevik et al., 2020), as well as intrinsic factor, which aids in absorption of vitamin B12 (Alpers and Russell-Jones, 2013), while the more basal chief cell secretes zymogenic proteolytic enzymes, including pepsinogen, (Raufman, 1992; Schubert, 2009), prochymosin (Richter et al., 1998), and gastric lipase (Moreau et al., 1989), which require acid activation. Together, these two epithelial lineages histologically and functionally define oxyntic mucosa (Rugge et al., 2008). In contrast, the more distal gastric antrum primarily functions to produce mucus, to regulate gastric acid secretion, and to propagate gastric peristalsis (O'Connor and O'Morain, 2014). These mucus-secreting glands differ in terms of morphology and composition compared to oxyntic glands of the corpus (Ban, 2013; Saenz and Mills, 2018). Antral glands are generally defined by a relative paucity of parietal cells and chief cells and by the presence of G cells (Watson et al., 2006), whose main product, gastrin, regulates acid secretion by parietal cells of the corpus and stimulates growth of oxyntic mucosa (Walsh, 1990). The differences in glandular composition of these two gastric compartments not only underlie their distinct functions in the stomach but also help to define some of the histologic changes that occur during chronic gastric injury (Rugge et al., 2008), which will be subsequently discussed in more detail. Importantly, the morphologic distinction between corpus and antral glands is less pronounced in humans (Choi et al., 2014) than in experimental animal models, such as mice.

It is worth noting that the functional organization of the stomach (i.e., acid-secreting corpus vs. mucus-producing antrum) can be explained by gastric specification during development (Kim and Shivdasani, 2016; Willet and Mills, 2016). We must consider that most of the developmental mechanisms behind human gastric corpus and antrum specification have been inferred from studies in mammalian and non-mammalian experimental models, and obvious limitations exist when extrapolating these findings to the human stomach. Regardless, one method of characterizing the distinct development of the corpus and antrum is by the regional pattern of transcription factor expression (Gracz and Magness, 2011; Kim and Shivdasani, 2016). In the mouse, the regions of the stomach that will become the gastric corpus and gastric antrum can both be characterized by the transcription factors *Sox2* (Que et al., 2007; Gracz and Magness, 2011; Willet and Mills, 2016) and *Gata4* (Jacobsen et al., 2002; Willet and Mills, 2016; Thompson et al., 2018), but the gastric antrum can be further defined by *Pdx1* expression, which is absent from the corpus (Offield et al., 1996; Nomura et al., 2005; Fujitani et al., 2006). While this is an oversimplified method of distinguishing corpus from antrum, it can serve as a molecular framework for distinguishing antral and corpus progenitors. The phenotypic effects that result from inhibiting or mis-expressing these transcription factors is beyond the scope of this review and has been succinctly discussed elsewhere (Kim and Shivdasani, 2016; Willet and Mills, 2016; McCracken K. W. and Wells J. M., 2017).

Importantly, the spatiotemporal expression of local epithelial and mesenchymal signals (McCracken K. W. and Wells J. M., 2017) and their effects on lineage specification within corpus and antral glands can now be more easily recapitulated using gastric organoid culture models (Dedhia et al., 2016). Gastric organoids generated *de novo* from human pluripotent stem cells have been engineered to form differentiated antral (McCracken et al., 2014) or corpus organoids (McCracken et al., 2017), and this has expanded our understanding of the signaling pathways that drive corpus versus antral regionalization. For example, these studies have implicated *HNF1B*, expressed in the early endoderm, in antral specification and identified novel corpus-specific markers, such as *IRX1/2/3/5* and *PITX1* (McCracken et al., 2017). In addition, transient inhibition of MEK signaling enhanced expression of genes specific to mature parietal cells (McCracken et al., 2017), a defining cell type in the corpus. Finally, and perhaps most interestingly, temporal activation of WNT/β-catenin signaling promoted the development of corpus-like organoids at the expense of antral organoids (McCracken et al., 2014; Broda et al., 2019). Taken together, these findings would point to independent and inter-dependent developmental pathways for the functional regionalization of the stomach.

The distinct developmental pathways that drive corpus and antrum specification become particularly relevant during chronic gastric injury, when these developmentally regulated morphologic and molecular distinctions can become blurred. We refer to these epithelial changes as metaplasia, which is broadly defined as a change of cells to a form that does not normally occur in the tissue in which it is found (Kumar et al., 2005). Indeed, chronic inflammation in the corpus can lead to morphologic changes such that corpus glands begin to histologically resemble antral glands, which are not normally present in the majority of the gastric corpus. These glandular changes in the corpus have taken on various names, including pseudopyloric metaplasia (Hebbel, 1949; Magnus, 1954) and pyloric metaplasia (Morrissey et al., 1983; Steer, 1984; Chang et al., 2001) to describe antral-like glands observed adjacent to healing ulcers in the corpus (or duodenum) or to more generally denote the extension of antral-type glands into the corpus in the setting of chronic Hp infection (Goldenring, 2018). In either case, the defining features of these “antralized” corpus glands include the gradual loss of parietal cells and chief cells, resulting in atrophy, as well as the expression of metaplastic markers, some of which are inherent to antral glands (Xia et al., 2004b; Rubio et al., 2009). In the context of chronic Hp infection, the “antralization” of the corpus refers to the proximal progression of a front of inflammation from the antrum into the corpus, leaving in its wake a span of antral-like glands (Xia et al., 2000; Xia et al., 2004a). From a clinical standpoint, this pattern of antralization is not arbitrary: various longitudinal endoscopic studies suggest that the pattern of antralization proceeds from the corpus-antrum transition proximally into the corpus along the lesser curvature (Graham, 2014). More importantly, this pattern of inflammation carries an increased oncogenic risk (Cassaro et al., 2000) and forms the basis for endoscopically assessing pre-cancerous risk through gastric mapping, as defined by the Sydney system (Guarner et al., 2003).

**FIGURE 1:**
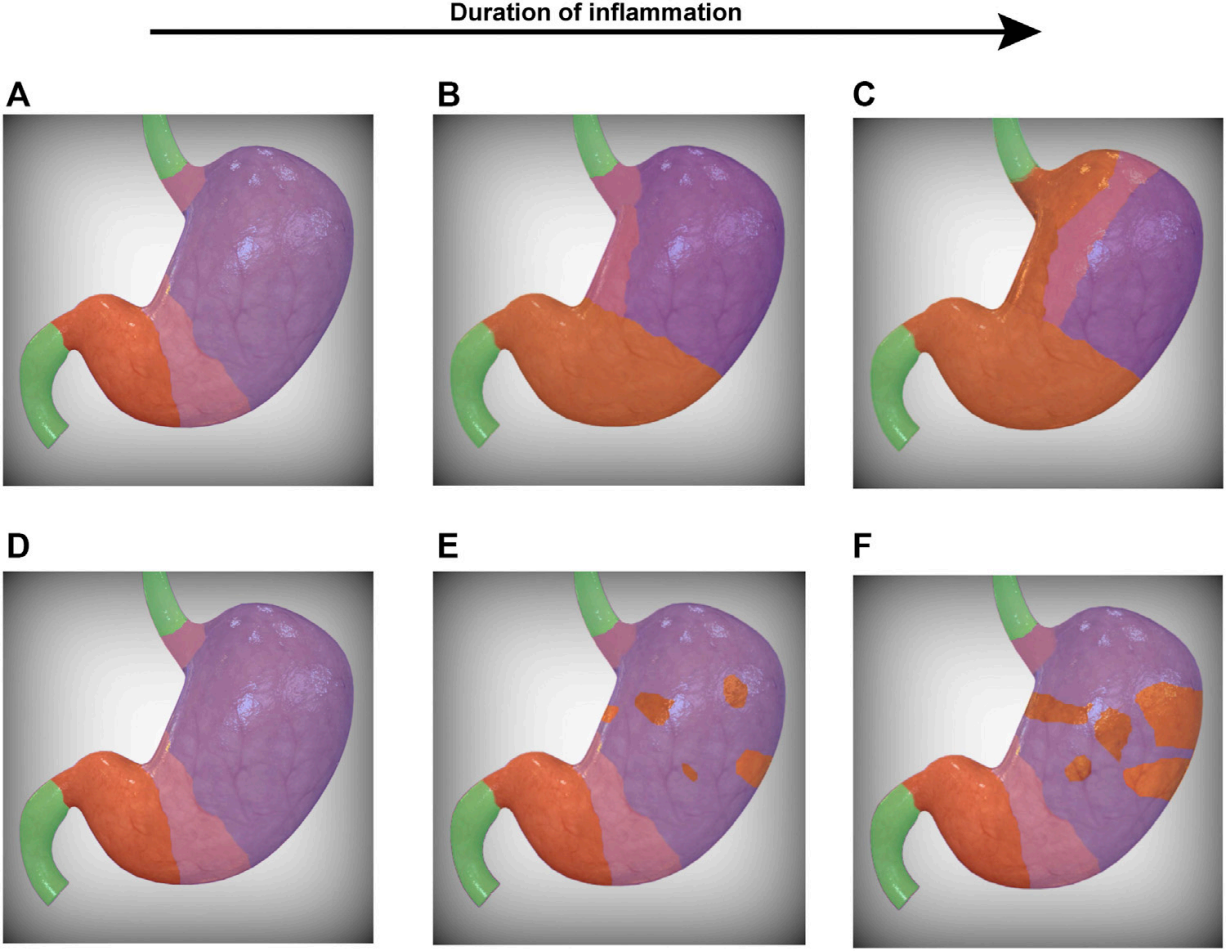
Progression of SPEM within the stomach. Two possibilities for the topographic progression of SPEM are presented. In **(A)**, the corpus-antrum and gastroesophageal transition zones (pink) represent foci from which SPEM arises. In the setting of chronic inflammation, the transition zones converge along the lesser curvature **(B)**, leaving in their wake gastric glands that morphologically resemble antrum (orange). The transition zone therefore represents a dynamic demarcation that serves as the leading edge for the progressive front of SPEM that ultimately extends into the greater curvature of the stomach **(C)**. Alternatively, SPEM may arise from multiple foci within the corpus **(E)**, gradually spreading radially and converging to form a lawn of SPEM **(F)**. It should be noted that these possibilities are not mutually exclusive and may occur simultaneously. SPEM, spasmolytic polypeptide-expressing metaplasia. The human stomach images were modified from a stock image purchased from turboquid.com.

It remains unclear why the antralization of the corpus follows a predictable expansion from the corpus-antrum transition and into the proximal corpus along the lesser curvature (Figures 1A–C). One could argue that, in the setting of chronic Hp infection, several foci of colonization could develop within the corpus and each give rise to a distinct nidus of inflammation from which atrophied (and perhaps antralized) glands can arise and/or radially expand in the setting of chronic inflammation (Figures 1D–F), though the evidence to date does not support this latter pattern. That the pattern of antralization instead emerges from the corpus-antrum border and extends along the lesser curvature, as opposed to the greater curvature, for example, suggests multiple possibilities that are not mutually exclusive. The first possibility is a programmed pattern of inflammation that develops irrespective of the insult: in the setting of chronic inflammation, the glands along the lesser curvature may adopt a more antral-like morphology. These glands may signal to neighboring glands to become more antral or to divide by gland fission, resulting in a gradual, gland-by-gland expansion. This possibility implies that antrum begets antrum and that the development of an antralized gland relies on an adjacent antralized gland. Another possibility relates to the span of antral glands that normally extends along the lesser curvature and into the fundus, with pyloric-type glands accounting for up to approximately 30% of the glands near the fundus in older individuals (Kimura, 1972). It is worth noting that the extension of the antrum into the corpus may occur with aging (Goldenring, 2018), though it appears to be accelerated and carry increased oncogenic potential in Hp-infected individuals (Umeda et al., 1971; Xia et al., 2002). It remains to be seen whether this strip of antral mucosa along the lesser curvature is a developmental remnant (Davis et al., 2017) and whether it serves as an area of heightened risk from which inflammation can arise (Busada et al., 2019).

Why, then, do injured corpus glands morphologically resemble antral glands, and does this suggest that the conversion to a more antral phenotype represents a programmed, default injury response in the corpus (Saenz and Mills, 2018)? Does injured corpus epithelium take on molecular features of antral epithelium, or is the metaplastic epithelial gene signature in the corpus unique? It is worth noting that the term “antralization” of the corpus (Xia et al., 2004a; Rubio et al., 2009) may not be the most appropriate description for the epithelial changes taking place within the chronically inflamed corpus, though alternative terms like pyloric or pseudo-pyloric metaplasia may be equally misleading (Goldenring, 2018). Injury to corpus glands is often characterized by a loss of acid-secreting parietal cells and chief cells, resulting in glands that morphologically resemble antral glands (Xia et al., 2000), but the similarities appear to end there. Following either acute or chronic injury, the corpus epithelium undergoes reparative changes and an expression of novel markers that are not typically expressed within the corpus at homeostasis. These cellular changes are consistent with the definition of metaplasia, which makes the term pyloric or pseudopyloric metaplasia somewhat appropriate, though these metaplastic markers, which continue to be inventoried (Weis and Goldenring, 2009; Weis et al., 2013; Weis et al., 2014; Meyer and Goldenring, 2018), are not simply a re-expression of antral (or pyloric) genes. Notably, these metaplastic glands lack gastrin-producing cells, or G cells, a defining feature of antral (or pyloric) glands (Dockray, 1999). Indeed, certain genes upregulated in metaplastic corpus epithelium are highly expressed in antral glands at homeostasis, including *DMBT1* (Sousa et al., 2012; Garay et al., 2017) and *GKN3* (Menheniott et al., 2010; Bockerstett et al., 2020), but by the same token, various genes appear to be unique to metaplasia in the corpus, neither expressed in corpus nor antral glands at homeostasis. These include genes such as *HE4* (Jeong et al., 2021; Nozaki et al., 2008; O'Neal et al., 2013), *CD44v9* (Fan et al., 1996; Bertaux-Skeirik et al., 2015; Bertaux-Skeirik et al., 2017; Tsugawa et al., 2019), and *CLU* (Weis et al., 2013; Shimizu et al., 2016)*,* among others (Weis et al., 2014). A more appropriate term to describe these glandular changes (and which will be used in this review to refer to these metaplastic changes) is spasmolytic polypeptide-expressing metaplasia (or SPEM), which is characterized by the expression of the antral spasmolytic polypeptide in the bases of corpus glands (Schmidt et al., 1999) and accurately describes the characteristic features of this metaplastic entity. Whether the expression of antralized or uniquely metaplastic genes increases the risk of neoplastic progression is largely correlative at this point. Regardless, chronically inflamed, metaplastic corpus glands adopt a hybrid expression pattern, demonstrating characteristics of antrum as well as features unique to the metaplastic corpus.

### Characterizing Metaplastic Phenotypes in the Stomach

The developmental demarcations that anatomically divide the stomach therefore appear to be fluid during chronic injury. It has become increasingly evident that injured gastric epithelium also undergoes a cellular reprogramming that is characterized, in part, by the re-expression of markers typically associated with a more fetal (or primitive) state (Mills and Sansom, 2015). These cellular changes can occur in post-mitotic, differentiated cells, underscoring an inherent plasticity within gastric epithelium in the face of acute or chronic injury. More importantly, these post-mitotic cells can also re-enter the cell cycle, often in an attempt to repopulate glandular cells that have been injured or lost. This stepwise cellular reprogramming, termed paligenosis (Willet et al., 2018; Miao et al., 2020), has been proposed as a potential mechanism by which cells can appropriately deal with injury. It is worth noting, however, that this process has largely been described in the zymogenic chief cell of the corpus (Willet et al., 2018; Burclaff et al., 2019), and it remains to be seen whether other gastric epithelial lineages, either in the antrum or corpus, rely on a similar reprogramming sequence or whether this even occurs under more indolent and less proliferative injury conditions (e.g., chronic Hp infection).

The reparative and metaplastic potential within epithelial cells of the corpus seems to be more lineage-restricted (Bjerknes and Cheng, 2002; Saenz and Mills, 2018), and most studies to date have focused on the isthmal stem cell (Hayakawa et al., 2015a; Hayakawa et al., 2017a, b; Karam and Leblond, 1993) and zymogenic chief cell in mice (Nam et al., 2010; Goldenring et al., 2011; Mills and Goldenring, 2017) as being capable for heeding the call for repair. More recently, a single-cell RNA-sequencing (scRNA-seq) and trajectory analysis looking at possible cellular origins of metaplasia found mucous neck cells and chief cells as putative SPEM precursors (Bockerstett et al., 2020). It is likely that the relative contributions of different epithelial lineages to metaplasia along the corpus gland axis can vary and are not mutually exclusive, based on the acuity and severity of the metaplasia-inducing injury. Future studies should continue to focus on this epithelial plasticity.

On the other hand, the epithelial cells lining antral glands have long been thought of possessing varying degrees of inherent stemness (Qiao et al., 2007; Barker et al., 2010; Hayakawa et al., 2015b). Recent scRNA-seq profiling of human antral biopsies (Zhang et al., 2019) would suggest that the ability of epithelial cells to become metaplastic and give rise to gastric pre-neoplastic lesions may not be as stochastic as previously thought, however. Within human antral glands, differentiated epithelial lineages can not only express unique metaplastic/stem cell markers but can also transcriptionally activate distinct response pathways within a given pre-neoplastic stage. As expected, the proportion of differentiated antral mucous cells, characterized by MUC6 and TFF2 expression, significantly decreased during the progression to gastric cancer and correlated with a rise in cells expressing intestinal stem cell transcripts, including *OLFM4*, *EPHB2*, and/or *SOX9*. However, during chronic atrophic gastritis, for example, pit mucous cells of the antrum were more likely to transcriptionally activate components of the metallothione pathway, while the deeper gastric mucous cells were more likely to express components of the TNF pathway. Moreover, certain epithelial lineages were more likely to acquire metaplastic or pre-neoplastic transcriptional signatures than others. For example, OLFM4 expression in intestinal metaplasia lesions was primarily found within the gastric mucous cells of the antrum, located deep within antral glands, compared to the surface pit cells (Zhang et al., 2019). Taken together, these findings would suggest that cellular reprogramming during injury is not uniform across cell types and that the development of pre-neoplastic lesions, within the corpus and antrum, may be lineage-restricted.

It should be noted that an additional metaplastic phenotype can arise in chronically inflamed gastric mucosa. The appearance of intestinalized epithelium, referred to as gastric intestinal metaplasia (GIM), represents a distinct pre-neoplastic gastric lesion that is believed to confer increased oncogenic risk (Cassaro et al., 2000; de Vries et al., 2008; Tan and Yeoh, 2015). GIM is often portrayed as succeeding oxyntic atrophy in the Correa cascade (Correa and Piazuelo, 2012), but this has never been formally proven (Goldenring et al., 2010). Regardless, the presence of GIM is often a surrogate marker of chronic inflammation, especially when detected in the gastric corpus (Choi et al., 2015; Graham et al., 2019). Gastric metaplasia characterized by intestinalized epithelium (either small intestinal or colonic) remains a bit of a pathologic enigma. For one, gastric mucosa is not the only mucosa that adopts an intestinal phenotype following chronic inflammation, as intestinal metaplasia, referred to as Barrett’s metaplasia/esophagus, can also be seen in the chronically inflamed distal esophagus and also marks mucosa at risk of progressing to cancer (Souza et al., 2008; Peters et al., 2019). GIM exhibiting histologic features of small intestinal epithelium, often referred to as complete GIM, is defined by small intestinal-type mucosa containing goblet cells, a brush border, and eosinophilic enterocytes, whereas incomplete GIM demonstrates colonic-type epithelium with irregular mucin droplets and the lack of a brush border (Correa et al., 2010). This latter histologic entity is believed to carry significant prognostic weight, as patients with incomplete GIM have been felt to be at a heightened cancer risk compared to those with complete GIM (Gupta et al., 2020). A recent study of a 20-year follow-up of Colombian patients found that those with incomplete GIM at baseline had an odds ratio of greater than 13 for developing gastric cancer compared to those with complete GIM at baseline (Piazuelo et al., 2021). These differences in neoplastic risk would imply the importance of histologic sub-typing of GIM, though additional longitudinal studies are warranted (El-Zimaity et al., 2001; Altayar et al., 2020; Gupta et al., 2020). Complete GIM also represents a more differentiated intestinal epithelium, complete with a brush border and the expression of acidic mucins, while the colonic epithelium of incomplete GIM may represent less differentiated, sialomucin-predominant cells (Correa et al., 2010). How the histologic characterization of these metaplastic subtypes translates to differences in risk has not been investigated mechanistically, however. Regardless, this would suggest that the conversion (or reversion) of chronically injured epithelium to an intestinal phenotype could be part of a conserved, metaplastic, and pre-neoplastic response.

It also remains unclear which injured cells give rise to intestinal cells in GIM. While there is more evidence for the cells that give rise to SPEM (Nam et al., 2010; Radyk et al., 2018), the cellular origin(s) and mechanisms of intestinalization in GIM are less clear. Some of the characteristic molecular features of human GIM have been described and shown to induce GIM when ectopically expressed in murine gastric mucosa. The homeobox gene *Cdx2* is central to intestinal differentiation in the developing gut, as well as to the maintenance of differentiated intestinal cells (Barros et al., 2010; Barros et al., 2012). CDX2 expression under the embryonically transcribed *Foxa3* promoter led to extensive intestinal metaplasia in the mouse stomach (Silberg et al., 2002), as did CDX2 expression under the parietal cell-specific *Atp4b* (β subunit of the H^+^/K^+^-ATPase) promoter (Mutoh et al., 2002). In addition, transgenic expression of the small intestinal transcription factor, CDX1, in parietal cells led to the replacement of oxyntic mucosa with intestinalized crypts with differentiated enterocytes, goblet cells, enteroendocrine cells, and Paneth cells. Interestingly, the location of Paneth cells was in the upper portion of the crypts, suggesting that cell-autonomous aspects of Paneth cell differentiation may not be fully recapitulated in this model. GIM in this mouse model was characterized by intestinalized cells scattered along the gastric gland axis, with the gland largely retaining gastric features and gastric epithelial lineages. Taken together, there appears to be evidence for CDX1/2, for example, in driving intestinal differentiation of undifferentiated cells of the foregut as well as inducing a trans-differentiation of adult parietal cells (Barros et al., 2012), but which of these cell fate mechanisms (if any) is pathologically relevant has yet to be determined. In the end, one could make an argument for both scenarios. On the one hand, injured gastric epithelium can take on a more fetal, progenitor-like state, and the expression of intestinal drivers, such as CDX1/2, in these reprogramming cells can direct them toward an intestinal cell type. On the other hand, intestinal cells can arise from the trans-differentiation of an adult gastric epithelial cell, similar to the trans-differentiation of chief cells to SPEM cells that has been reported (Weis and Goldenring, 2009; Meyer and Goldenring, 2018). One could speculate that all epithelial injury represents a transient reversion to a more primitive, progenitor state, and the spatiotemporal expression of certain lineage-defining features in this malleable population of cells dictates their fate.

### Clinical Implications of Gastric Atrophy and SPEM

At a more general level, what are the pathophysiologic implications of these changes in gastric landscape, both at the anatomic and glandular levels? Clinically, the anatomic extent of SPEM serves as a surrogate for the degree and duration of inflammation (Graham et al., 2019). It is generally assumed that the extent of atrophy and SPEM within the corpus portends a poorer clinical prognosis than atrophy restricted to the antrum (Hebbel, 1949; El-Zimaity, 2006). The corpus-restricted atrophy associated with autoimmune gastritis is a unique case (Neumann et al., 2013) and will not be discussed in this review. For patients with evidence of chronic inflammation, the pattern of atrophy and SPEM throughout the stomach is endoscopically mapped for purposes of risk stratification and surveillance. However, recent surveillance guidelines (Gupta et al., 2020) that rely on endoscopic mapping using the Sydney system (Dixon et al., 1996) focused on the histologic identification and mapping of GIM. Typically, these islands of GIM arise in a sea of atrophy and SPEM (Goldenring et al., 2010). GIM’s patchy distribution can make accurate mapping and surveillance all the more difficult (Crafa et al., 2018; Waddingham et al., 2018). Indeed, the presence of GIM in the gastric corpus is believed to confer increased oncogenic risk (Cassaro et al., 2000), but the guidelines for the timing and frequency of endoscopic surveillance of GIM in the United States remain vague (Gupta et al., 2020) and require further study.

Perhaps a more reasonable approach would be to consistently map the pattern of atrophy and SPEM, regardless of GIM. Whether GIM evolves from atrophic mucosa and independently confers increased oncogenic risk remain unresolved (Graham and Zou, 2018), but GIM in the corpus is almost invariably surrounded by atrophic mucosa (Goldenring et al., 2010). Importantly, the extent of atrophy has been shown to correlate with the risk of progressing to cancer (Liu et al., 2005). One Japanese study found that patients with severe (i.e., extensive) atrophy at index endoscopy were more likely to develop gastric cancer than patients with mild or moderate atrophy, within the time period studied (Shichijo et al., 2016). One could argue that endoscopically mapping the extent of corpus atrophy (or antralized corpus) would therefore be a more reliable and equally effective method for risk stratification than inconsistent sampling of patchy GIM (Kotelevets et al., 2021). Of note, this is commonly practiced in the Far East (Kishino et al., 2016; Song et al., 2017) using the Kimura-Takemoto classification (Kimura, 1969), which relies on the endoscopic pattern of atrophy to risk stratify patients. In addition, mucosal atrophy, unlike GIM, may still represent a reversible pathologic state (Rokkas et al., 2007; Lee et al., 2013), where Hp eradication can lead to a regression of atrophy or at the very least a halt in the progression to cancer. Identifying the extent of atrophic (or antralized) mucosa also seems more consistent with the pattern of field lineage changes that occur as a result of chronic inflammation and that follow a predictable, proximal extension from the antrum into the corpus along the lesser curvature, as discussed previously.

### How SPEM Affects Hp Pathogenesis

The extent of atrophy and SPEM has equally important implications in terms of understanding Hp colonization. Rather than focusing on how Hp effects metaplastic changes in the colonized stomach, one should consider how these metaplastic changes affect Hp pathogenesis. Hp has been shown to hone in on injured gastric epithelium, as seen in ulcerated mucosa. While injured mucosa may express chemoattractants for Hp (Aihara et al., 2014; Huang et al., 2015; Huang et al., 2016), ulcer margins are lined by SPEM glands that are believed to facilitate mucosal repair (Teal et al., 2020). Recent evidence suggests that Hp has a tropism for SPEM glands, and this is in part mediated by binding of the SabA adhesin to its cognate receptor, sialyl-Lewis X (sLe^x^), which shows enhanced expression in metaplastic glands (Mahdavi et al., 2002; Saenz et al., 2019). More importantly, the expression of sLe^x^ extends deeper into the bases in SPEM glands (Saenz et al., 2019), allowing Hp to burrow deep within these glands. Teleologically, this could allow Hp not only to establish an acid-protected niche but also facilitate Hp’s interactions with proliferating, metaplastic cells near the gland base (Saenz and Mills, 2018). As Hp has been shown to inject genotoxins and induce distinct patterns of DNA damage (Hartung et al., 2015; Bauer et al., 2020), the interactions of Hp with actively dividing, metaplastic cells could be an under-appreciated method by which Hp promotes pre-neoplastic changes.

The pattern of atrophy and metaplasia throughout the corpus mimics the pattern of Hp’s spread throughout the stomach. The gradual migration of Hp from the antrum and into the corpus likely progresses along the lesser curvature (Van Zanten et al., 1999; Graham, 2014). It remains unclear, however, whether Hp is actively driving this pattern of SPEM through a gland-by-gland migration into the corpus, or whether Hp is simply following the pattern of SPEM glands, which are the result of chronic inflammation and for which Hp has an increased affinity (Mahdavi et al., 2002; Saenz et al., 2019). Regardless of this chicken vs egg scenario, it seems that Hp exploits the very metaplastic changes in the corpus that it has induced. This becomes particularly important when we consider the initial establishment of Hp infection and its clinical implications to the host. If we consider that Hp has a tropism for metaplastic glands, then we can speculate that metaplastic glands will be targeted and preferentially colonized upon initial infection.

**FIGURE 2:**
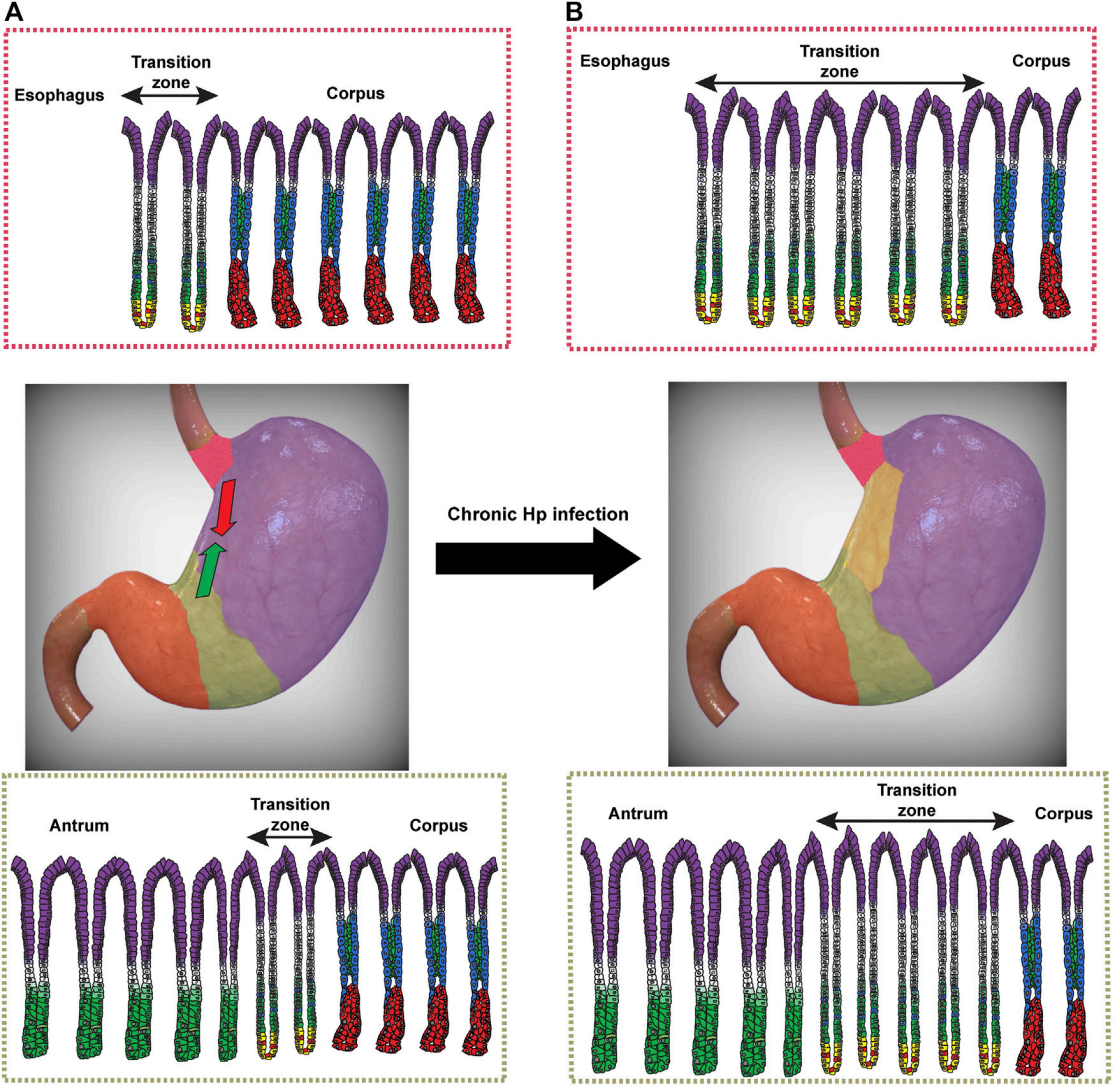
Expansion of transition zones during chronic *Helicobacter pylori* (Hp) infection. **(A)** Transition zones exist between the distal esophagus and glandular corpus (red) and between the corpus and antrum (green). At homeostasis, corpus glands are characterized by acid-secreting parietal cells (blue) lining the gland axis and zymogenic chief cells at the gland base (red). Pit cells (purple), mucous neck cells (green), and the proliferative isthmal stem cells (white) are also shown. At homeostasis, the cardia **(top)** contains a small number of glands within the transition zone (double-sided arrow) that show a mixed mucous/oxyntic morphology, with a relative paucity of parietal cells and an expansion of SPEM cells (yellow) with hybrid features of chief cells and mucous neck cells. A similar transition zone exists between the glands of the corpus and antrum **(bottom)**. **(B)** Following chronic Hp infection, the number of glands with mixed mucous/oxyntic morphology increases, and the transition zones expand bi-directionally to converge along the lesser curvature. The human stomach images were modified from a stock image purchased from turboquid.com.

Interestingly, at baseline and prior to the expansion of Hp-induced metaplasia, two anatomic and histologic junctions exist within the uninjured stomach and may represent hot spots for Hp colonization (Van Zanten et al., 1999). The first junction occurs at the transition from corpus to antrum, with a span of multiple glands with mixed features of both mucous and oxyntic glands (Odze and Goldblum, 2009; Stave et al., 1978). These glands show a decrease in parietal cell number per gland, as one moves distally toward the antrum (Grossman, 1960); Figure 2A). This likely represents a developmental transition in glandular composition from parietal- and chief cell-dense “pure” corpus glands to antral glands, which contain lower densities of these lineages. It also theoretically represents a transition in acidity (Engel et al., 1984; Schade et al., 1994), though local acid levels in this transition zone have not been accurately determined. Whether the spread of Hp into the corpus is simply a result of alterations in gastric acidity or changes in the metaplastic landscape remains an open question, but the transition zone likely serves a critical role both for the expansion of metaplasia in the host and for Hp pathogenesis (Van Zanten et al., 1999). Indeed, in Hp-infected patients with intact acid secretion, the pattern of gastritis is largely restricted to the antrum, where Hp likely avoids the inhospitable local acidity of the corpus and can more effectively replicate (Saenz and Mills, 2018). Over time, Hp-induced chronic inflammation can lead to a more proximal, corpus gastritis, ultimately leading to a loss of parietal cells and a hypo- or achlorhydric state, thereby allowing Hp to more effectively colonize the corpus (Bayerdorffer et al., 1989; Fiocca et al., 1992). The effect of lowering gastric acidity on Hp-induced gastritis was elegantly shown over 25 years ago, when Kuipers and others demonstrated that Hp-infected patients on chronic acid suppression, in the form of PPIs, were more likely to develop corpus gastritis than Hp-infected patients not on acid suppressants (Kuipers et al., 1996). This would argue that gastric acidity is a major determinant of Hp’s ability to colonize the corpus, as has been demonstrated in the murine stomach (Danon et al., 1995; Saenz et al., 2019). However, we would equally argue that, in addition to changes in gastric acidity that can accompany chronic inflammation, Hp can induce a characteristic metaplastic landscape, as discussed earlier, that it can exploit and follow in order to migrate out of the antrum and expand its niche. Indeed, this corpus-antrum transition zone has been shown to represent an area of consistent and persistent inflammation in infected patients (Genta et al., 1994b). We would agree with previous speculation (Van Zanten et al., 1999) that this transition zone serves as a nidus from which metaplasia, and subsequently Hp colonization, can expand proximally into the corpus.

A similar transition zone exists between the distal esophagus and proximal stomach, often referred to as the cardia in humans (or limiting ridge in mice). Like with the corpus-antrum transition, there exists a span of mixed mucous/oxyntic glands as one moves from the distal esophagus to the gastric corpus (Figure 2A; Odze, 2005). The significance of the cardia in Hp pathogenesis remains largely understudied. Some studies have noted that the gastric cardia was one of the most consistently colonized regions of the stomach in Hp-infected patients, as determined endoscopically (Genta et al., 1994a; Liao et al., 2018). It remains to be seen whether the cardia indeed represents a preferential site for initial Hp colonization, like the corpus-antrum transition. Taken together, however, these transition zones could represent dynamic hot spots for the establishment of Hp infection. As Hp is able to delve deeper into glands with lower densities of parietal cells (Saenz et al., 2019), these transition zones could represent safe havens for Hp to colonize and replicate while avoiding the harsh acidic gastric lumen in the surface epithelium.

### Transition Zones as Hot Spots for Gastric Tumorigenesis

In addition to their contribution to Hp colonization and spread, the transition zones at the cardia and corpus-antrum border may also represent areas of increased oncogenic potential. Similar to the regional specification of the glandular stomach discussed previously, the delineation between the esophagus and the stomach at the gastroesophageal junction arises from a gradient of transcription factor expression as well as from communication between the mesenchyme and endoderm (Vogt and Panoskaltsis-Mortari, 2020). For example, *Hoxa5* expression in the mesenchyme of murine embryos stimulates patterned expression of the transcription factors Sonic hedgehog (*Shh*) in esophageal epithelium and Indian Hedgehog (*Ihh*) in columnar gastric epithelium (McCracken K. W. and Wells J. M., 2017). In addition, the gastroesophageal junction is also defined by the coordinated development of muscle fibers of the lower esophagus that form the lower esophageal sphincter. The proper development and orientation of the smooth muscle fibers in the lower esophagus depend on *Cdo*, which encodes for a co-receptor for Hedgehog signaling. In the absence of *Cdo*, smooth muscle fascicles in the lower esophagus do not properly align (Romer et al., 2013).

Histologically, the gastric cardia is defined as the mucosa that is distal to the anatomic gastroesophageal junction and proximal to the characteristic oxyntic glands of the gastric corpus (Odze, 2005). There exists a debate on the origin and development of the gastric cardia, and it relies in part on whether the presence of mixed mucous/oxyntic glands is a result of a metaplastic conversion of pure corpus glands from chronic injury, such as gastroesophageal reflux disease (GERD) (Chandrasoma et al., 2000; Chandrasoma et al., 2003), or whether these mixed glands are normally present from birth (Kilgore et al., 2000; Glickman et al., 2002; Derdoy et al., 2003). While the intent of this review is not to engage in this debate, the correlation between the mixed glands that characterize this transition zone and the presence of cardia gastric tumors may not be coincidental.

Anatomically, the cardia can be exposed to various inflammatory insults, be it recurrent exposure to acid (Chandrasoma, 2005) and/or colonization by Hp (Genta et al., 1994a), as previously discussed. The etiology of inflammation of the gastric cardia, or carditis, can be distinguished histopathologically, with GERD-induced carditis showing more eosinophils in the inflammatory infiltrate and Hp-induced carditis demonstrating a more dense infiltrate of plasma cells, for example (Wieczorek et al., 2003; Odze, 2005). However, distinguishing whether a tumor in the cardia arose from metaplastic glands of the distal esophagus, gastric corpus, or cardia, can be more difficult (Wolf et al., 2001; Spechler, 2004). While various epidemiologic studies suggest that chronic Hp infection does not appear to increase the risk of cardia gastric cancer (Helicobacter and Cancer Collaborative, 2001; Abdi et al., 2019), tumorigenesis in the gastric cardia may be more nuanced. In the setting of chronic Hp infection, cancers arising in the cardia were positively associated with glandular atrophy and hypochlorhydria (Hansen et al., 2007), features consistent with non-cardia gastric cancer. But some studies have also suggested that the hypo- or achlorhydria that can develop from chronic Hp infection may protect against cancer of the cardia by reducing gastric acidity (Kamangar et al., 2006). In patients with chronic Hp infection and extensive atrophy involving the corpus, it is possible that atrophy and metaplasia begin in the cardia, an area of consistent Hp colonization and gastritis (Genta et al., 1994a). In the setting of chronic inflammation, this focus of atrophy and metaplasia migrates distally from the cardia into the corpus. That extensive atrophy can be seen in certain patients with cancer of the gastric cardia (Hansen et al., 2007) would simply illustrate the severity and duration of inflammation, but we should not lose sight of the fact that this atrophy and metaplasia may have originated in the cardia and that the glands of the cardia would have sustained the longest duration of inflammation and metaplastic injury. Whether the mixed mucous/oxyntic glands of the cardia are inherently at an increased risk of becoming metaplastic is unknown, but this transition zone appears to be at a unique risk for metaplastic injury and neoplasia.

The transition zone at the corpus-antrum junction may represent an area with similar metaplastic risk as the gastric cardia. Unlike cardia gastric cancer, the risk of non-cardia gastric cancer seems to be more strongly associated with chronic Hp infection and not directly linked to GERD (Axon, 2002; Moss and Malfertheiner, 2007). However, like the cardia, this junction is composed of a span of mixed mucous/oxyntic glands that are frequently colonized by Hp and where gastritis is consistently observed (Figure 2A; (Bayerdorffer et al., 1992; Satoh et al., 1991). As a result, this transition zone may also represent the initial site from which Hp and the metaplastic front can migrate proximally into the corpus. One could therefore imagine a bidirectional progression of metaplasia, moving (perhaps synchronously) distally from the cardia and proximally from the corpus-antrum junction and converging along the lesser curvature (Figure 2B). This pattern of atrophy and metaplasia carries an increased risk of progressing to cancer relative to other endoscopic patterns of metaplasia (Cassaro et al., 2000). This pattern of progression along the lesser curvature also represents the most direct route for the convergence of these two migrating, dynamic transition zones.

More importantly, the corpus-antrum transition zone, like the cardia, represents a region of sustained inflammation and metaplastic injury during chronic Hp infection. The duration of regional inflammation during chronic Hp infection should not be minimized, as these areas are the most prone to accumulating oncogenic mutations, either as a result of cellular proliferation during metaplastic reprogramming (Saenz and Mills, 2018) or from direct Hp-induced genotoxic injury (Hartung et al., 2015; Bauer et al., 2020). This may explain the fact that most non-cardia gastric adenocarcinomas are anatomically located in the antrum (You et al., 1992; Wanebo et al., 1993), even though the antrum occupies a significantly smaller surface area than the corpus. If Hp preferentially colonizes the antrum and the mixed mucous/oxyntic glands of the corpus-antrum junction when establishing an infection, then these regions would be exposed to inflammation longer than the corpus, which primarily experiences inflammation after Hp has migrated proximally (Bayerdorffer et al., 1989). Whether gastric cancers that arise solely in the corpus are related to initial Hp colonization and sustained gastritis in this region is not known. Regardless, the histologic characteristics of these transition zones, coupled with what we currently understand about Hp pathogenesis, could explain the patterns of atrophy, metaplasia, and cancer in chronically infected patients.

## Conclusion

While we have enhanced our understanding of some of the topographic and glandular changes that result from chronic inflammation, we still have a rudimentary grasp of where gastric pre-neoplastic lesions first arise and why certain patterns of atrophy and metaplasia increase oncogenic risk. The transition zones (esophagus/gastric corpus and gastric corpus/antrum) may represent epicenters from which atrophy and SPEM develop and spread, but fundamental questions remain. What role(s), if any, do these poorly defined regions play in gastric tumorigenesis? Developmentally speaking, how are these transition zones formed and maintained, and are they inherently metaplastic? Do similar transition zones contribute to patterns of pathology and tumorigenesis seen in other chronically inflamed organs? As our appreciation of the mechanisms that give rise to atrophy and SPEM continue to evolve, we are likely to find that the functional and regional demarcations in the stomach, though developmentally determined, are fluid during injury and highlight an inherent plasticity in an organ that must constantly regenerate and adapt to injury.
